# Tilapia Head Protein Hydrolysate Attenuates Scopolamine-Induced Cognitive Impairment through the Gut-Brain Axis in Mice

**DOI:** 10.3390/foods10123129

**Published:** 2021-12-17

**Authors:** Jun Ji, Xiangzhou Yi, Yujie Zhu, Hui Yu, Shuqi Huang, Zhongyuan Liu, Xueying Zhang, Guanghua Xia, Xuanri Shen

**Affiliations:** 1 Hainan Engineering Research Center of Aquatic Resources Efficient Utilization in South China Sea, Hainan University, Haikou 570228, China; jijunbest@126.com (J.J.); xiangzhouyi1995@hainanu.edu.cn (X.Y.); zyj199711117@163.com (Y.Z.); yuhui19970607@163.com (H.Y.); hsq00603@163.com (S.H.); liuzhongyuan999@126.com (Z.L.); 994257@hainanu.edu.cn (X.Z.); xiaguanghua@vip.163.com (G.X.); 2 College of Food Science and Technology, Hainan University, Haikou 570228, China; 3 Collaborative Innovation Center of Marine Food Deep Processing, Dalian Polytechnic University, Dalian 116000, China

**Keywords:** tilapia head protein hydrolysate, cognitive impairment, oxidative stress, gut-brain axis, cholinergic system, gut microbiota

## Abstract

The destruction of the homeostasis in the gut-brain axis can lead to cognitive impairment and memory decline. Dietary intervention with bioactive peptides from aquatic products is an innovative strategy to prevent cognitive deficits. The present study aimed to determine the neuroprotective effect of tilapia head protein hydrolysate (THPH) on scopolamine-induced cognitive impairment in mice, and to further explore its mechanism through the microbiota–gut-brain axis. The results showed that THPH administration significantly improved the cognitive behavior of mice, and normalized the cholinergic system and oxidative stress system of the mice brain. The histopathological observation showed that THPH administration significantly reduced the pathological damage of hippocampal neurons, increased the number of mature neurons marked by NeuN and delayed the activation of astrocytes in the hippocampus of mice. In addition, THPH administration maintained the stability of cholinergic system, alleviated oxidative stress and further improved the cognitive impairment by reshaping the gut microbiota structure of scopolamine-induced mice and alleviating the disorder of lipid metabolism and amino acid metabolism in serum. In conclusion, our research shows that THPH supplementation is a nutritional strategy to alleviate cognitive impairment through the gut-brain axis.

## 1. Introduction

According to statistics, there are about 50 million people suffering from dementia worldwide, and it is estimated that the number of patients will increase to 131 million by 2050, which will bring a huge burden to society and families [1,2]. Alzheimer’s disease (AD) is the most common manifestation of dementia, and its clinical manifestation is progressive cognitive dysfunction [3]. Typical symptoms of this disease include cholinergic system defects, oxidative stress, inflammation, hippocampal neuronal death, neurofibrillary tangles, and amyloid plaques [4]. Among them, the cholinergic hypothesis is one of the theories that explain the pathogenesis of AD. Studies have shown that the brains of patients with AD show cholinergic system disorders, including the relative loss of cholinergic neurons, the reduction of acetylcholine (Ach) level and the number of acetylcholine receptors (AchR), which is also one of the reasons leading to decreased learning ability and memory defects [5,6]. In addition, uncontrolled oxidative stress during neurodegeneration unbalanced the cholinergic system and causes brain injury, eventually leading to cognitive impairment [7,8,9]. This indicates that maintaining the homeostasis of the cholinergic system and inhibiting the development of oxidative stress are of great significance in the prevention of cognitive deficits.

Despite years of research, effective pharmacological therapy for cognitive impairment related to prodromal AD and mild AD dementia remains a major unmet need in clinical practice. Currently, three drugs based on cholinergic pathways in the central nervous system for the treatment of AD-associated dementia, donepezil, galantamine, rivastigmine, which have been approved by the US Food and Drug Administration (FDA), have anticholinesterase activity, but their long-term safety and efficacy utility are not entirely clear [10], and the newly approved drug aducanumab in 2021 also has a lot of controversy about its side effects [11]. Clinical trials have shown that these approved drugs either had poor efficacy or limited improvement in early AD and were not suitable for long-term administration [12]. Therefore, before seeking an accurate monotherapy strategy for cognitive impairment, we shift to diet intervention for disease prevention. Nowadays, the protein hydrolysates represent a good strategy to enhance the health of animals; indeed, the diet administration in animals model has been accepted as a useful approach to promote the formation of highly antioxidant peptides to contrast oxidative stress [13]. Many studies have also shown that neurodegenerative diseases could be prevented by changing the daily diet through its effect on the microbiota–gut-brain [14,15,16]. This will further contrast oxidative stress in the body of animals or human, and dietary composition is important for the structure and balance of gut microbiota. However, the abnormal changes in the structure of gut microbiota, such as *Lactobacillus*, *Bifidobacterium* and *Prevotella* sp. led to the development of neurodegenerative diseases [17,18,19,20]. In addition, gut microorganisms are also considered to contribute to the energy metabolism, serum metabolism, and brain health of the host through microbial metabolites, such as short-chain fatty acids (SCFAs), secondary bile acids, amino acids, and neurotransmitters [21,22]. Other studies have shown that some microorganisms have the ability to produce acetylcholine to maintain the stability of cholinergic in the host body, thereby improving cognitive impairment [23]. Therefore, prevention of cognitive impairment through dietary intervention is feasible and easy-to-implement strategy.

It is well known that natural-derived functional substances have safer efficacy than synthetic chemical drugs and are harmless to the body after being long-term taken as daily diet, which further improve the subhealth state of the body [24]. Some bioactive peptides have demonstrated the ability to improve cognitive impairment and protect nerves [25,26]. Recent studies have found that peptides improved neurodegenerative diseases through the gut-brain axis [27]. For instance, peptide PW5 derived from walnut alleviated cognitive impairment by regulating gut microbiota and changing the acetylcholine level of serum [28]. Fish heads contain a large number of bioactive substances, including fish oil and peptides [29]. We evaluated the functionality of the fish head residue after oil extraction, which laid a theoretical basis for the high-value utilization of fish head byproducts. The peptides obtained from tilapia head were hypothesized to have potential neuroprotective activities, but their efficacy in improving cognitive impairment, and the main mechanism had not been specifically elucidated. As a result, we proposed a nutritional strategy for dietary intervention of tilapia head protein hydrolysate (THPH) to explore its neuroprotective effects on the brain and its mechanism.

In this study, the effects of dietary THPH supplementation on the cognitive behavior and memory disorders in mice through the scopolamine-induced cognitive impairment model were evaluated. Additionally, we determined the role of the microbiota–gut-brain axis in mediating THPH to improve cognitive impairment by multi-omics analysis of gut microbiota and serum metabolomics of mice, and we analyzed the potential mechanism of THPH to improve scopolamine-induced cognitive impairment. This study could provide a new high-value utilization method for tilapia processing byproducts and explore a potential functional food to prevent cognitive impairment.

## 2. Materials and Methods

### 2.1. Chemicals

Tilapia (*Oreochromis niloticus*) heads were provided by Hainan Xiangtai Fishery (Hainan, China). The alkaline protease and papain were purchased from Shanghai Yuanye Biotechnology Co., Ltd. (Shanghai, China) and Biofroxx (neoFroxx GmbH, Einhausen, Germany), respectively. Malondialdehyde (MDA), superoxide dismutase (SOD), reduced glutathione (GSH) and catalase (CAT) detection kit were purchased from Nanjing Jiancheng Bioengineering Institute (Jiangsu, China). Enzyme-linked immunosorbent assay (ELISA) kits for acetylcholine (Ach), acetylcholine receptor (AchR), choline acetyltransferase (ChAT) and acetylcholinesterase (AchE) were purchased from Meilian Biotechnology (Shanghai, China). Other chemical reagents used in this study were of analytical grade.

### 2.2. Preparation of THPH

Fresh tilapia heads were thawed at 25 °C, then we cleaned and removed the gills. The wet fish heads were put into the grinder until it became a paste. Then, ethanol was mixed with the paste with a ratio of 10:1 (*v*/*m*) and subsequently were sonicated (1.5 h for three times, 250 W) to remove most of the fat. After filtration, the fish head residues were dried at 55 °C for 12 to 14 h and finally grinded to obtain defatted tilapia head powder.

Tilapia head powder was initially dispersed in deionized water (1:8, *w*/*w*), and after high-speed homogenization (10,000 rpm, 2 min), it was dissolved in high-pressure steam (0.11 MPa, 121 °C) for 30 min. Subsequently, alkaline protease (protease/substrate 2%, *w*/*w*) was added for hydrolysis at pH 10.0, 55 °C for 5 h, and then papain (protease/substrate 2%, *w*/*w*) for hydrolysis at pH 6.5, 55 °C for 5 h. After the enzymolysis, the hydrolysate was treated at 95 °C for 20 min to inactivate the enzyme, and then centrifuged (8000 rpm, 4 °C, 20 min). The supernatant was collected and lyophilized to obtain tilapia head protein hydrolysate (THPH).

### 2.3. Determination of Amino Acid Composition and Molecular Weight (MW) Distribution of THPH

The amino acid composition was determined according to the method of Mei et al. [30]. In short, THPH were hydrolyzed with 6 M HCl at 110 °C for 24 h and amino acid analyses were performed using HPLC (Agilent 1100, Agilent Ltd., Santa Clara, CA, USA). Amino acid standard solutions were used as external standards and the contents of the individual amino acids were expressed as g/100 g protein.

The MW distribution of THPH were measured following the method of Chen et al. [31]. Briefly, a Waters (Waters Corporation, Milford, MA, USA) 1525 HPLC system with a silica-based column (TSK-GEL G2000 SWXL, Shimadzu, Tokyo, Japan) was used for the analysis. Samples were continuously eluted with the mobile phase at a flow rate of 0.5 mL/min and monitored at 214 nm. Four protein standards, cytochrome c (12,400 Da), bacitracin (1450 Da), Gly-Gly-Tyr-Arg (451 Da), and Gly-Gly-Gly (189 Da) were used to make standard curve.

### 2.4. Animals Research

#### 2.4.1. Animals

A total of 36 SPF male BALB/c mice (20 ± 2 g, aged 6–8 weeks) were purchased from Hunan Slack Jingda Animal Experiment Co., Ltd. (Changsha, China, Certificate number: SCXK (Xiang) 2019-0004). The mice were assigned under a suitable environment with a controllable temperature of 22 ± 1 °C and 55 ± 5% relative humidity as well as a 12 h light/dark cycle per day. All mice were allowed to move freely in their cages and were provided with sufficient distilled water and standard maintenance feed containing more than 18% protein (Hunan Jiatai Experimental Animal Co., Ltd., Hunan, China, Permit number: SCXK (Xiang) 2020-0006) and weighted every two days during the experiment.

#### 2.4.2. Dosage Regimen

**Figure 1:**
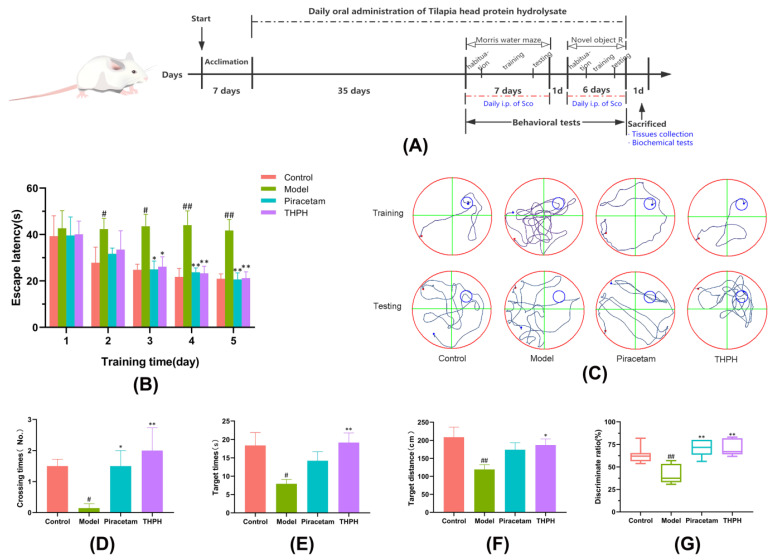
Effect of THPH on the behavioral performance of the Morris water maze test and the novel object recognition test in scopolamine-induced amnesia mice. (**A**) Experimental protocol of animal tests. Sco indicates scopolamine; i.p. indicates intraperitoneal injection. (**B**) Effect of THPH on escape latency of mice in the training trials. (**C**) Representative swimming paths of mice on the fifth day of the training period and the testing day. (**D**) Number of times the mice crossed the platform on the testing day. (**E**) Swimming times of the mice in the target quadrant on the testing day. (**F**) Swimming distance of mice in target quadrant on the testing day. (**G**) Discrimination index of mice in novel object recognition test. Data are presented as means ± SEM. ^#^ *p* < 0.05 and ^##^ *p* < 0.01 compared to the control group; * *p* < 0.05 and ** *p* < 0.01 compared to the model group.

After adaptive feeding for 7 days, the mice were randomly divided into four groups throughout the animal experiments (*n* = 9 in each group), including control (normal mice), model (scopolamine with 4.0 mg/kg body weight), piracetam and THPH groups. The mice in the control group and model group were initially administrated with physiological saline orally at 1 mL/100 g body weight every day for 35 days. For piracetam and THPH groups, piracetam (400 mg/kg body weight) and THPH (400 mg/kg body weight) with 1 mL/100 g body weight were given a gavage every day for 35 days, respectively. Subsequently, the mice in the model, piracetam and THPH groups were intraperitoneally injected with scopolamine 4 mg/kg at 30 min before the start of the behavioral experiment, while the control group mice were injected with saline. Behavioral tests were examined for 14 days, including 7 days of Morris water maze test and 6 days of novel object recognition experiment, as well as one day of relaxation period. The whole experiment lasted for 56 days, and the mice were euthanized on the 57th day (Figure 1A). The brain, blood and feces were collected for analysis.

#### 2.4.3. Morris Water Maze Test

The Morris water maze test was performed based on previous research methods with minor modifications [32]. Briefly, the water maze was a circular pool divided into four quadrants and filled with water, and an invisible escape platform was located 1 cm below water in one quadrant. In the place navigation trial, the mice were slowly put into the water facing the pool wall, and the monitoring time of swimming was set as 60 s. The computer acquisition system documented the swimming path, escape latency and movement distance of mice. The effective escape latency was the time for mice to reach the platform within 60 s and stay there for 3 s, while the escape latency of mice that failed to locate the platform within 60 s was recorded as 60 s. Subsequently, in the space probe trial, the escape platform was removed and the number of platform crossings, the target quadrant time (swimming time in the target platform quadrant) and the target quadrant distance (swimming distance in the target platform quadrant) within 60 s were recorded.

#### 2.4.4. Novel Object Recognition Test

After the end of water maze test and rest for 1 day, all mice were subjected to novel object recognition test for 6 days. Novel object recognition test was performed based on the previous research method with slight modifications [33,34]. Briefly, in the first two days of the experimental test, each mouse was allowed to explore the field of plexiglass box for 5 min to adapt to the environment. Then in the training phase, the box was divided into four areas, and two identical objects (A and A’) were placed in the center of the diagonal area. The mice were put into the field from the center of the box after intraperitoneal injection for 30 min and allowed to explore objects freely for 5 min. After each test, the objects and instruments were cleaned with 75% ethanol. During the last day of testing, a new object was used to replace one of the two identical objects in the training, and the new object was similar in size but different in shape and color. The time spent by the mice exploring the new object and the familiar old object was recorded respectively within 5 min. Exploratory behavior was defined as a mouse using its nose or front paws to smell and touch an object, but the mouse sitting on the object or turning around was not considered an exploration behavior. The result was expressed as discrimination index (DI), which was the time taken to explore a new object divided by the total time taken to explore the old and new two objects, of which 50% was considered incidental. The higher discrimination index indicated preferable object recognition memory.

#### 2.4.5. Collection of Tissue, Blood and Feces

The mice were sacrificed and dissected on ice, brain tissues and blood were collected. Part of brain tissues were fixed with 4% (*v*/*v*) paraformaldehyde solution for further histopathological and immunofluorescence staining analysis. The remaining brain tissues were immediately frozen in liquid nitrogen. Blood samples were centrifuged (3000 rpm, 4 °C, 20 min) to separate the serum, and fresh mouse feces was collected in sterile enzyme-free tubes. The brain, serum, and feces were stored at −80 °C until analysis.

### 2.5. Biochemical and Histopathological Analysis in Mouse Brain

#### 2.5.1. Determination of Biochemical Parameters

The mouse brain tissues were homogenized with PBS (pH 7.4) at 4 °C and centrifuged (12,000 rpm, 4 °C, 20 min) to obtain the supernatant. The levels of AchE, ChAT, Ach, and AchR were measured using ELISA kits guided by the manufacturer’s protocol. In addition, the activity of SOD and CAT, and the content of GSH and MDA were measured according to the commercial kits.

#### 2.5.2. Histopathological and Immunofluorescence Analysis

Nissl staining was performed to assess the morphological changes of neuronal cells in the hippocampus of the mouse brain. The fixed brain tissues were dehydrated with ethanol and embedded in paraffin. Sections were cut into 5 μm by the microtome, and stained with cresyl violet. The microscopic images of the mouse hippocampus were obtained with a Nikon microscope (NIKON Eclipse ci, Tokyo, Japan), and the morphological structural changes of the mouse hippocampal neurons were observed at different magnifications.

The immunofluorescence staining was performed based on previous research methods with slightly modified [35]. The brain tissue sections (5 μm) were stained with antibodies to detect NeuN and GFAP. After antigen repaired with EDTA antigen retrieval buffer (pH 8.0), the tissue sections were blocked with 5% bovine serum albumin (BSA) for 30–40 min. The sections were incubated with anti-NeuN and anti-GFAP at 4 °C overnight. Subsequently, the sections were incubated with the secondary antibody at 24 °C for 50 min before counterstaining the sections with 4’,6-diamidino-2-phenylindole (DAPI). Finally, the expression of related proteins in the mouse hippocampus was imaged with a NIKON ECLIPSE CI microscope (Nikon, Tokyo, Japan).

### 2.6. Quantitative Reverse Transcription-Polymerase Chain Reaction (qRT-PCR)

The qRT-PCR experiment was performed basing on the previously reported methods with slight modification [36,37,38]. In brief, total RNA was extracted from brain tissues homogenized of mice by Eastep^R^ Super Total RNA Extraction Kit (Promega Biotechnology Co., Ltd., Shanghai, China). One microgram of purified RNA was used for RT-PCR to synthesize cDNA with RevertAid First Strand cDNA Synthesis Kit (Thermo Fisher Scientific, Waltham, MA, USA). The obtained cDNA was then used for quantitative polymerase chain reaction on a real-time PCR detection system (Bio-Rad, Hercules, CA, USA). The relative expression level of mRNA (relative to the control group) was measured by the 2^-ΔΔCt^ method with GAPDH as the internal reference. The primer sequences used in this experiment were as follows:

- AchE (forward)-5′-AGCAATATGTGAGCCTGAACCTGAAG-3′;
- AchE (reverse)-5′-CTCCGCCTCGTCCAGAGTATCG-3′;
- ChAT (forward)-5′-ATTGGGTCTCTGAATACTGGCTGAATG-3′;
- ChAT (reverse)-5′-TGGTCATTGGTGTCTTGGAAGTGC-3′;
- GAPDH (forward)-5′-AAGAAGGTGGTGAAGCAGGCATC-3′;
- GAPDH (reverse)-5′-CGGCATCGAAGGTGGAAGAGTG-3′.

### 2.7. Microbial DNA Extraction, PCR Amplification and High-Throughput Sequencing in Feces

To determine the expression of the gut microbiota flora associated with cognitive impairment, 16s high-throughput sequencing was used to study the changes in the intestinal flora. The method of genomic DNA amplification was the same as in the previous research [38]. Briefly, the microbial DNA in mouse feces was extracted using QIAamp DNA Stool Mini Kit (Qiagen, Hilden, Germany), and then the purity and concentration of DNA were detected by agarose gel electrophoresis. The 16s rDNA V3-V4 region of the ribosomal RNA gene was amplified by PCR. The PCR products were detected by 2% agarose gel electrophoresis, and the amplicons were extracted from 2% agarose gels, purified using the QIAquick PCR purification kit (Qiagen, Germany), and then the amplicons were quantified. Finally, sequencing analysis and species identification were carried out for the purified amplicons.

### 2.8. Untargeted Metabolomics Profiling in Serum

Seven serum samples from each group were selected for untargeted metabolomics analyses. The samples (100 μL) were placed in the EP tubes and mixed with 80% methanol and 0.1% formic acid. Then the samples were centrifuged at 15,000× *g*, 4 °C for 20 min after vortexed and incubated on ice for 5 min. Some of supernatant was diluted with LC-MS grade water to a solution with final concentration of methanol of 53%. After centrifugation (15,000× *g*, 4 °C, 20 min), the supernatant was injected into the LC-MS/MS system (Thermo Fisher, Germany) for untargeted metabolomics analysis.

### 2.9. Statistical Analysis

Excluding the above statistics of the gut microbiome and serum metabolome, data from other experiments were expressed as mean ± SEM (standard error of the mean). The data between each group were compared and analyzed by SPSS 26.0 statistical software (IBM, Armonk, NY, USA) with one-way ANOVA and the LSD multiple comparison test. For all analyses, *p* values less than 0.05 and 0.01 were considered to indicate significant and extremely significant differences, respectively.

For 16s rDNA high-throughput sequencing, a small fragment library was constructed according to the characteristics of the amplified 16S rDNA V3-V4 region, and then paired-end sequencing (Paired End) was performed on this library based on the Illumina NovaSeq sequencing platform. After stitching and filtering Reads and OTUs (Operational Taxonomic Units) clustering, species annotation and abundance analysis were used to reveal the composition of the sample’s gut microbes.

For serum untargeted metabolomics, metabolites were annotated using the KEGG database, the HMDB database and the Lipidmaps database. Cluster analysis was performed on the different metabolites between the comparison groups. The metabolite data was normalized using z-score, and the cluster heat map was drawn by the Pheatmap package in R language. The functions of these metabolites and metabolic pathways were studied using the KEGG database.

## 3. Results

### 3.1. Amino Acid Composition and MW Distribution of THPH

Amino acid composition and MW distribution played an important role on the activity of protein hydrolysates. Among them, the low MW peptides had its potential physiological activity [39]. As illustrated in Table 1, the content of MW > 3 kDa fraction in THPH accounted for only 1.48%, while the fraction with MW lower than 3 kDa was 98.52% in THPH, and more than 60% of the peptides were less than 500 Da. As shown in Table 2, the highest level of Glu was observed, followed by Gly and Pro in the hydrolysates. Hydrophobic amino acids in THPH accounted for 29.58%, in which the contents of Pro, Ala, and Leu were high. Notably, high levels of Glu and hydrophobic amino acids might have a positive effect on the neuroprotective of THPH.

### 3.2. Effect of THPH on the Behavioral Test of Mice Induced by Scopolamine

#### 3.2.1. Effect of THPH on Spatial Memory of Scopolamine-Induced Mice in the Morris Water Maze

Spatial memory was considered as one of the most basic manifestations of higher cognition in mammals, including the capacity to record and store information and spatial orientation recognition [40,41]. As shown in Figure 1B, the enhancement of the spatial memory capacity of each mouse was manifested by the shortening of the escape latency over successive days of training. During the training period, the escape latency of the scopolamine-induced mice was not changed significantly, which indicated that the mice in the model group cannot accurately find the platform through training. The data in the last two days of the training period showed that the escape latencies of mice in the THPH group and the piracetam group significantly decreased compared to the model group. As showed in Figure 1C, compared to the control group (20.91 ± 5.14 s), the swimming track of the model group mice was disorganized and aimless (41.73 ± 11.50 s), while the THPH treatment group mice had a clear purpose of finding the platform (21.20 ± 6.66 s).

Similarly, in the spatial probe trial, we observed that the scopolamine-induced mice reduced the number of crossing the target platform, the swimming time and distance in the target quadrant after the platform was removed (Figure 1D–F). The swimming time of the THPH-given mice in the target quadrant increased significantly from 7.93 ± 2.94 s to 19.18 ± 6.49 s (*p* < 0.01), and the swimming distance increased significantly from 119.23 ± 35.55 cm to 187.05 ± 42.03 cm (*p* < 0.05). Additionally, supplementation of piracetam and THPH significantly increased the number of times that the mice crossed the platform, compared to the model group. Furthermore, we observed from Figure 1C that mice in the model group swam aimlessly and barely reached the position of the original platform, while the mice that had been fed THPH seemed to have remembered the location of the original platform, so they had been hovering in this quadrant. As expected, mice treated with THPH showed improvement in cognitive and memory abilities in the Morris water maze.

#### 3.2.2. Effect of THPH on Recognition Behavior of Novel Object in Scopolamine-Induced Mice

Novel object recognition tasks were used to evaluate the recognition behavior of nonspatial memory [42]. As shown in Figure 1G, the recognition index of the mice in the scopolamine-treated group was less than 50%, indicating that the time for the mice in this group to recognize new object was less than old object. The mice fed piracetam and THPH took more time to recognize new objects, which indicated that the ability of the mice to recognize novel objects had increased significantly (*p* < 0.01). This result further verified that pre-administration of THPH prevented cognitive deficits in mice induced by scopolamine and improve their recognition ability.

### 3.3. Effect of THPH on the Cholinergic System and Oxidative Stress in the Brain of Mice

#### 3.3.1. Effect of THPH on Ach, AchR, ChAT and AchE Levels in the Brain

**Figure 2:**
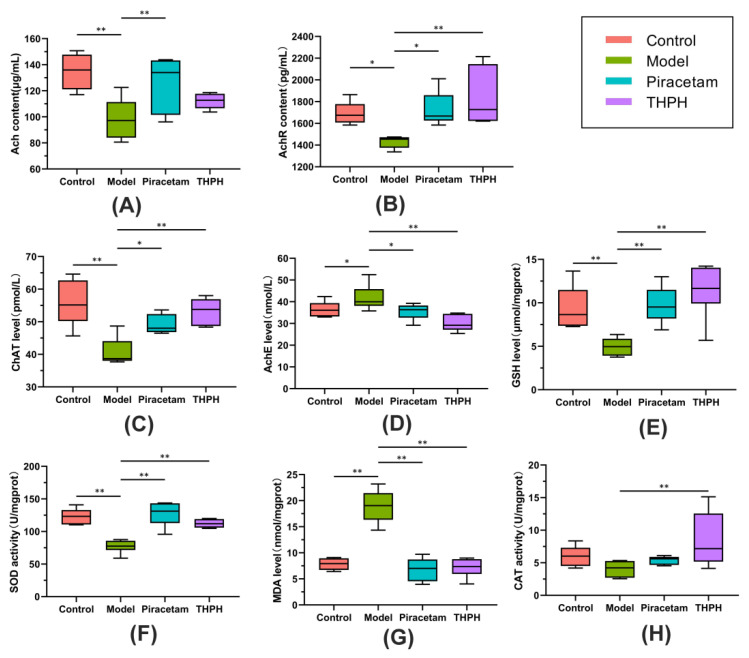
Effect of THPH on the cholinergic system and the oxidative stress in the brains of scopolamine-induced amnesia mice. Cholinergic system: (**A**) Level of Ach. (**B**) Level of AchR. (**C**) Level of ChAT. (**D**) Level of AchE. Oxidative stress: (**E**) Level of GSH. (**F**) Activity of SOD. (**G**) Level of MDA. (**H**) Activity of CAT. Data are presented as means ± SEM. * *p* < 0.05 indicates significant difference and ** *p* < 0.01 indicates extremely significant difference.

Dysfunction of the cholinergic system plays a crucial factor in patients with low cognitive ability, and the level of the neurotransmitter Ach in the brain is closely related to cognitive function. The levels of AchE, ChAT, Ach and AchR were shown in Figure 2. The levels of Ach, AchR and ChAT in the model group were significantly lower than those in the control group (Figure 2A–C), and the AchE level significantly increased (Figure 2D). Simultaneously, compared to the model group, the levels of AchR and ChAT in the THPH group significantly increased to 29.56% and 30.67%, respectively, and the level of AchE significantly decreased to 28.03%. In addition, the contents of Ach, AchR and ChAT in piracetam group also increased, while the level of AchE decreased significantly.

#### 3.3.2. Effect of THPH on Oxidative Stress Parameters in the Brain

The oxidative stress system plays a very important role in learning and memory. To evaluate the effects of THPH on the regulation of the oxidative stress system in the brain of cognitive impairment mice, we detected the parameters such as GSH, SOD, MDA and CAT, which were closely related to oxidative stress. The results showed that compared to the control group, the level of GSH and the activity of SOD in model group significantly reduced to 47.29% (Figure 2E) and 37.35% (Figure 2F), respectively. The level of MDA significantly increased (Figure 2G). However, there was no significant change of CAT between the model group compared to the control group (Figure 2H, *p* > 0.05). We observed that the level of GSH and the activities SOD and CAT in the THPH group significantly increased compared to the model group (Figure 2E,F,H). The content of MDA recovered to a similar level of the control group after THPH treatment (*p* < 0.01). Similarly, supplementation of piracetam also reversed these symptoms other than CAT (Figure 2).

### 3.4. Effect of THPH on the Hippocampus of Mice with Cognitive Impairment by Histopathological Staining

#### 3.4.1. Neuronal Protective Effects of THPH in the Hippocampus of Mice

**Figure 3:**
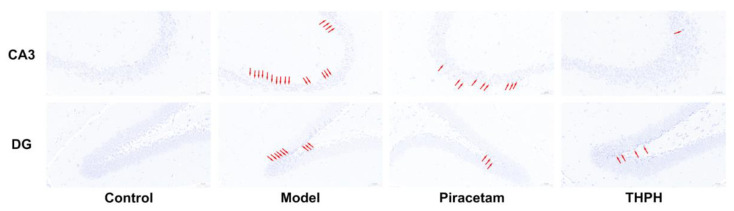
Effect of THPH on morphological changes of neurons in CA3 and DG regions of hippocampus in scopolamine-induced amnesia mice. Histological changes were examined by Nissl staining. The arrows indicate the damaged neurons. Scale bar is 50 μm.

The morphological changes of neurons in the hippocampus of the brain are closely related to the function of learning and memory, which includes four regions: CA1, CA2, CA3 and the dentate gyrus (DG). The histological features of the hippocampus of mice in each group were shown in Figure 3. Compared to the control group, the cells arrangement in the hippocampal CA3 and DG region of mice in the model group were disordered and loose, while the cytoplasm reduced and the cell nucleus was blurred, and there was a large area of nuclear pyknosis and pyramidal cell necrosis. Notably, THPH treatment significantly reversed the neuronal damage in hippocampal CA3 and DG regions, and showed tighter cell arrangement and less neuronal cell damage. Specifically, the morphology of the hippocampal cells in the THPH group was regular, the nucleus and cytoplasm were clearly visible, and the nuclear pyknosis was also significantly reduced. In addition, the neuronal damage in the hippocampus of the mice in the piracetam treatment group was also improved.

#### 3.4.2. THPH Increased the Neuronal Population and Decreased Reactive Astrocyte Clusters

**Figure 4:**
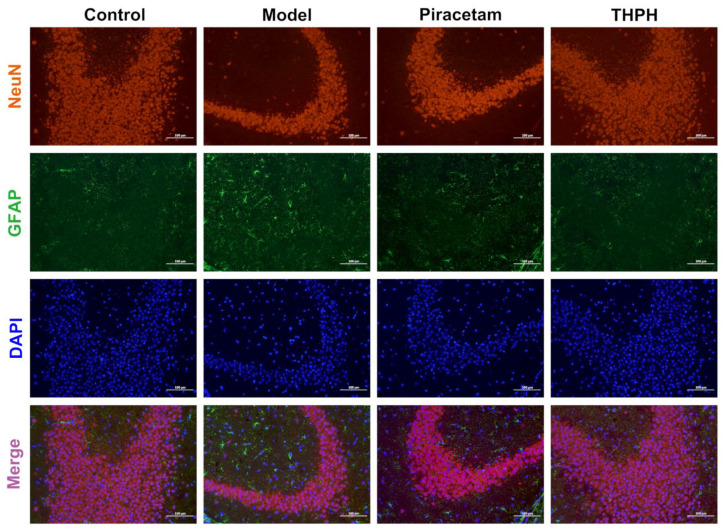
The neuroprotective effects of THPH on increasing the number of mature neurons and modulating activated astrocyte clusters in the hippocampal CA3 region of scopolamine-induced amnesia mice. Immunostaining for NeuN (red) to mark mature neurons, GFAP (green) to mark activated astrocytes in the hippocampal CA3 region of each group, and DAPI (blue) to mark the nucleus of living cells, Merge (purple) represents the superposition of the above fluorescence, scale bar is 100 μm.

To further determine the protective effect of THPH on neurons, we next examined whether THPH treatment promote neurogenesis, which includes proliferation, differentiation and survival of neurons. As shown in Figure 4, there were fewer NeuN positive cells (the marker of mature neuron) in the CA3 region of the mice hippocampus in the model group. However, in the THPH-treated mice, the number of NeuN-positive cells in the hippocampal CA3 region significantly increased, indicating that THPH treatment had a positive effect on neurogenesis. Meanwhile, a similar situation appeared in the piracetam group. In addition, we observed extensive GFAP positive cells (the marker of astrocyte activation) in the scopolamine-treated group (Figure 4). Fortunately, after THPH treatment, the GFAP positive cells significantly decreased, indicating that THPH effectively prevented the proliferation of activated astrocytes and might reduce oxidative stress and neurotransmitter level disorder.

### 3.5. Effect of THPH on the mRNA Expressions of AchE and ChAT in the Brain

**Figure 5:**
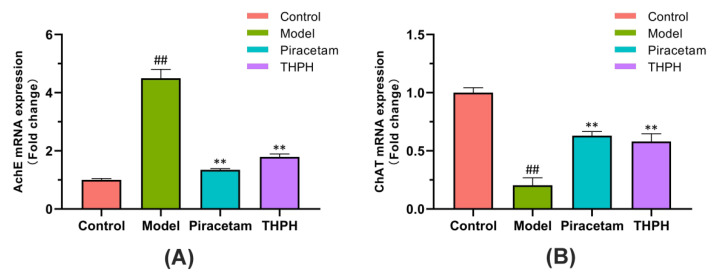
Effect of THPH on the mRNA expressions of (**A**) AchE and (**B**) ChAT in scopolamine-induced amnesia mice. Data are presented as means ± SEM. ^##^ *p* < 0.01 compared to the control group; ** *p* < 0.01 compared to the model group.

We further clarified THPH’spossible mechanism of improving cognitive impairment and memory by studying the expression of AchE and ChAT genes in the brain. The mRNA expression of AchE in the model group was more than four times higher than that in the control group (Figure 5A), but the mRNA expression of ChAT significantly reduced (Figure 5B), and the expression of AchE and ChAT genes was significantly reversed following treatment with piracetam or THPH (Figure 5).

### 3.6. Effect of THPH on Gut Microbiome Composition

**Figure 6:**
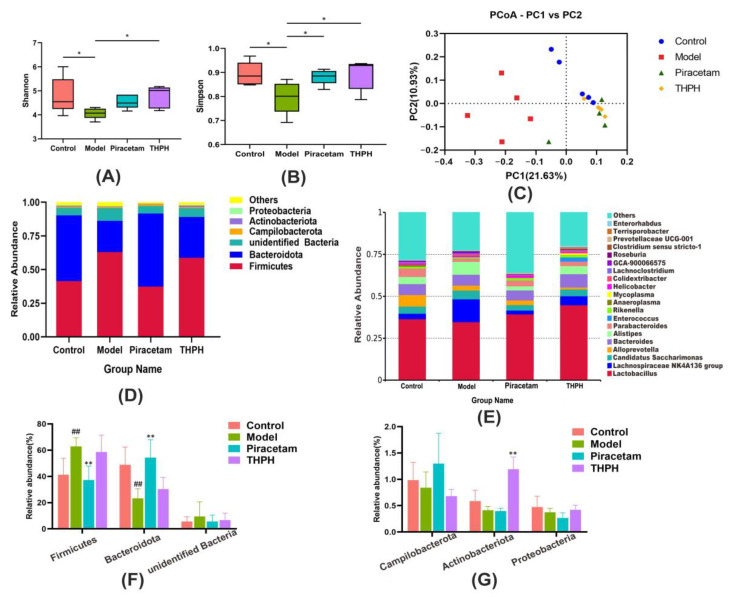
Effect of THPH supplementation on gut microbiota structure in scopolamine-induced amnesia mice. Feces microbiota composition was analyzed by 16S rRNA gene sequencing. (**A**) Shannon index in α-diversity. (**B**) Simpson index in α-diversity. (**C**) Principal coordinate analysis (PCoA) based on Unweighted Unifrac distance in β–diversity. (**D**) Relative abundance at the phylum level of each group. (**E**) Relative abundance at the genus level of each group. (**F**) Relative abundance of *Firmicutes, Bacteroidota* and *unidentified* *Bacteria* at the phylum level. (**G**) Relative abundance of *Campilobacterota, Actinobacterota* and *Proteobacteria* at the phylum level. Data are presented as means ± SEM. * *p* < 0.05 indicates significant difference in (**A**,**B**). ^##^ *p* < 0.01 compared to the control group; ** *p* < 0.01 compared to the model group.

Functional ingredients in animal and plant foods played their role in improving cognitive impairment by regulating the gut microbial community and diversity [43,44,45]. The results showed that the Shannon index and Simpson index of the α diversity significantly reduced in the model group compared to the control group (Figure 6A,B), while those in the THPH group significantly increased. It indicated that the THPH diet reversed the decrease in the abundance and uniformity of intestinal microbes in mice caused by scopolamine treatment. Regarding β-diversity, principal coordinate analysis (PCoA) was performed based on the Unweighted Unifrac distance. The results showed that the model group was significantly separated from the other three groups, indicating that the community structure of intestinal microorganisms in mice induced by scopolamine was obviously changed. It is worth to note that after THPH diet intervention, its community structure was brought back to a similar level as that in the control group (Figure 6C).

**Figure 7:**
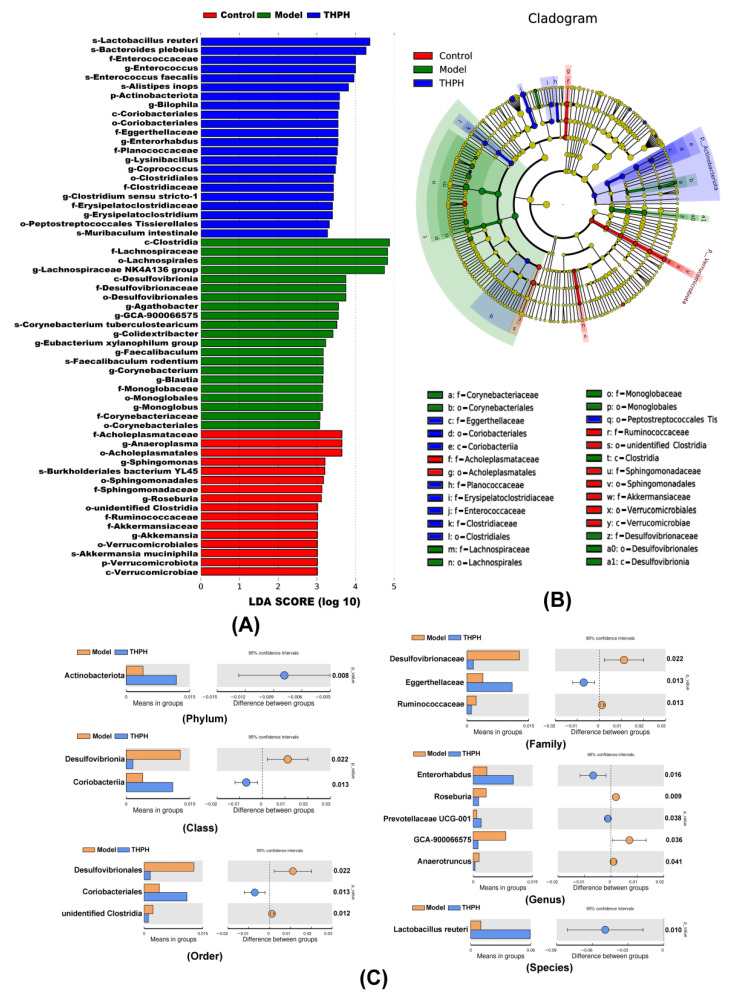
Effect of THPH on the significantly different bacteria in gut microbiota. (**A**) Linear discriminant analysis (LDA) of the gut microbiota in different groups. (**B**) Cladogram generated from linear discriminant analysis (LDA) showing the relationship between taxon. (**C**) Significantly different bacteria abundance maps between the model group and the THPH group at different taxonomic levels obtained by *t*-test.

To further illustrate the changes of gut microbiota after THPH diet, we performed analysis of microbial communities at different levels. *Firmicutes*, *Bacteroidota*, *unidentified Bacteria* and *Campilobacterota* were dominant bacteria (Figure 6D), and 20 main genera were found in all groups (Figure 6E). As showed in Figure 6F,G, compared to the control group, the relative abundance of *Firmicutes* significantly increased in the model group, and the relative abundance of *Bacteroidota* obviously decreased, while the other phyla showed no significant changes. The piracetam group relatively reversed the colony changes caused by the model group. However, we found that the relative abundance of *Actinobacteriota* significantly increased in THPH group with a slightly decrease of Firmicutes and a slight increase of *Bacteroides*. Subsequently, the LDA Effect Size (LEfSe) was applied to further determine the specific individual bacterial communities differentially enriched between different groups. The results showed that 59 biomarkers with an LDA score higher than 3.0 (Figure 7A). Scopolamine-treated mice showed a significant change in the levels of 21 bacteria, including two classes, four orders, four families, nine genera and two species. In addition, 22 bacteria were significantly enriched in the THPH group, including *Lactobacillus reuteri, Bacteroides plebeius,* and others (Figure 7B). Simultaneously, we further found that THPH mainly upregulated the abundance of *Actinobacteriota*, *Prevotellaceae UCG-001*, *Lactobacillus reuteri* and downregulated the abundance of pathogenic bacteria such as *Desulfovibrionia* through *t*-test (Figure 7C). These results indicated that THPH significantly altered the gut microbiome of mice with cognitive impairment induced by scopolamine.

In addition, we predicted the potential functional interactions of metabolic pathways between gut microbiota and hosts in different dietary groups, which were shown in the cluster heat map (Figure S1). It was seen that the THPH group had significant impact on human diseases, followed by environmental information processing and genetic information processing. Furthermore, in the second-level KEGG pathways (Figure S1), THPH supplementation was associated with nine microbial function metabolic pathways. Among them, the levels of signaling molecules and interaction and metabolism of other amino acids were abundant.

### 3.7. Effect of THPH on Serum Metabolites

THPH diet demonstrated a certain impact on gut microbiota, raising our interest in investigating whether and how it affected serum metabolites. Multivariate statistical analysis identified differences in serum metabolomics among the control, model, and THPH groups. Overall, the untargeted metabolite profiling of serum samples revealed that 75 differentially expressed endogenous metabolites (31 upregulated and 44 downregulated) were obtained in the model group compared to the control group (Figure S2), and 133 metabolites showed significant changes after THPH treatment (Figure S3). Specifically, THPH effectively restored 17 serum metabolites of cognitive impairment mice to the levels of the control group, including lysophosphatidylcholine 16:1, N-acetyl-1-aspartylglutamic acid, etc. In addition, THPH also increased the serum levels of taurine, L-histidine, choline, taurochenodeoxycholic acid and decreased the level of corticosterone in serum.

In order to further understand the functional characteristics and classification of the different metabolites in these three groups, KEGG was applied to conduct pathway enrichment analysis. We found that scopolamine caused disorders in lipid metabolism and amino acid metabolism in mice serum, including linoleic acid metabolism, phenylalanine metabolism, and alanine, aspartate and glutamate metabolism (Figure S4). However, after THPH supplementation, several serum metabolic pathways were significantly changed, including β-alanine metabolism, neuroactive ligand-receptor interaction (Figure S4). In addition, we also noticed changes in other metabolic pathways in the THPH group, such as the cysteine and methionine metabolism as well as taurine and hypotaurine metabolism.

## 4. Discussion

A large number of studies have shown that active protein peptides derived from natural plants and animals have many biological characteristics and wide functions, which also play a positive role in improving cognitive impairment [46]. However, whether THPH could alleviate the cognitive impairment and the role played by the related gut microbiota and serum metabolites were still unclear. Thus, this study has found for the first time that supplementation of THPH alleviated cognitive impairment and enhanced memory via the microbiota–metabolites–brain axis.

The stronger biological activity of low MW peptides has been widely reported [47]. The fraction with the MW less than 3 kDa, which was obtained by double-enzyme digestion, has reached 98.52% in THPH. Moreover, the results of amino acid composition showed that THPH contained the high levels of Glu and hydrophobic amino acids. Glu was an excitatory neurotransmitter, which was beneficial to the brain repairment of mice with cognitive impairment [48]. These results implied that THPH had neuroprotective effects. Furthermore, we intuitively demonstrated the reversal effects of THPH on scopolamine-induced cognitive impairment through two behavioral experiments. To our knowledge, the Morris water maze test is an important, even dominant, method used to evaluate spatial mapping versus working memory; it is usually used to assess the rodent spatial learning and memory or reference memory [40]; the novel object recognition test is a simplified task based on the innate curiosity of rodents for the assessment of cognitive changes in mice or rats, which is mainly used to evaluate the recognition memory on objects or environments in mice [49,50]. In both tasks, the mice showed excellent spatial memory ability and object recognition ability after THPH treatment, which were deduced from the existence of special amino acids, polypeptides and unique structural characteristics of THPH.

Furthermore, the morphological changes of neurons in the hippocampus were assessed through histopathology, demonstrating that THPH-treatment alleviated cellular damage, including disordered arrangement and neuronal necrosis in the CA3 and DG regions. A large number of studies have shown that there are significantly low levels of expression of NeuN and high-level activation of astrocytes in the brain of mice or humans with cognitive impairment, especially in the hippocampus, which will further reduce hippocampal neurogenesis, which is consistent with our findings [51,52,53]. We observed in Figure 4 that NeuN positive cells significantly increased in the hippocampal CA3 region of the THPH-treatment mice, which indicated that THPH restored damaged neurons to normal and made positive effects on neuronal proliferation, thereby further improving brain damage and achieving neuroprotective effects. Several recent studies have also shown that the proliferation and activation of astrocytes render neuronal constituents more susceptible to oxidative damage, and further lead to brain diseases, such as neurodegeneration or some neurodevelopmental disorder [54,55,56]. Fortunately, we found that THPH treatment effectively prevented this phenomenon from happening (Figure 4), which provided further evidence for THPH to improve cognitive impairment. In addition, our study showed that THPH administration significantly reduced the oxidative stress response in brain of mice with cognitive impairment induced by scopolamine, including markedly increasing the activities of SOD and CAT, raising GSH level, and reducing the content of MDA. Previous studies had shown that low MW peptides effectively alleviated the oxidative stress caused by scopolamine, which was consistent with our findings [57,58]. This was deduced from the presence of many low MW (<3 kDa) peptides and abundant highly hydrophobic amino acids in THPH, which made it easier to cross the cell membrane to reach the target site and scavenge excess free radicals and reactive oxygen species (ROS), thereby protecting brain cells from oxidative injury.

The development of oxidative stress led to dysfunction of cholinergic system, which would cause dysfunction of nerve signal transmission, further impairing memory function [59]. Under the normal conditions of the cholinergic system, Ach is synthesized by choline and acetyl-CoA through ChAT and released by vesicles into the synaptic cleft. After Ach was released, it bound to AchR on the postsynaptic membrane and then transmitted signals such as memory in the brain. Therefore, downregulating the level of AchE and upregulating the levels of ChAT, Ach and AchR are important ways to maintain the balance of cholinergic system. The results after THPH treatment were generally consistent with this assumption. Moreover, our results also found that the changes in the levels of the two enzymes (AchE and ChAT) that determined Ach level were consistent with their gene expression. The transcriptomics results showed that THPH diet reduced the production of AchE by inhibiting AchE mRNA over-expression, and they promoted the expression of ChAT mRNA to restore its level. These results indicated that THPH alleviated the cholinergic system disorder caused by scopolamine, which further improved the cognitive impairment and enhancing memory.

The disorder of the microbiota–gut-brain axis affected the cholinergic system and oxidative stress system in the brain, further leading to the occurrence of neurodegenerative diseases [60]. Our study revealed strong links between the alterations of gut microbiota and the changes of serum metabolome profile and the indicators related to the cholinergic system and oxidative stress system in THPH diet mice by integrated analysis of multi-OMICs data. Previous studies had found that peptides prevented cognitive impairment by improving the composition of the gut microbiota and its metabolites [61]. As we expected, this study found that THPH administration altered gut microbiota diversity in cognitive impairment mice, including increasing the abundance of *Actinobacterota*, which produced metabolites with anti-AchE activity [62]. THPH treatment also increased the abundance of probiotics, such as *Lactobacillus reuteri* and *Prevotellaceae UCG-001*, which had the effects of regulating neural behavior and improving cognitive function, and produced metabolites that participated in the hosts’ lipid metabolism to maintain the brain function [63,64,65,66]. In addition, THPH reduced the severity of gut microbiota dysbiosis by altering the structure of the microbial community via the decreased abundance of pathobionts, including *Desulfovibrionales*, *Desulfovibrionaceae*, and *Anaerotruncus*, which related to cognition and neurodegenerative diseases. Furthermore, we predicted that other amino acids metabolism related to metabolic pathways of gut microbiota changed. Taken together, these results suggested that the abnormality of gut microbiota was involved in the pathogenesis of scopolamine-induced cognitive impairment. Moreover, THPH diet ameliorated the abnormal gut microbiota, which provide therapeutic targets for cognitive dysfunction.

The THPH-restructured gut microbiota led to alterations in the microbial metabolites in serum, which further explained the mitigation effect of THPH on cognitive impairment in mice. Our study found that the serum amino acid metabolism and lipid metabolism of mice with cognitive impairment were disordered, which was consistent with the results of a previous clinical trial [67]. The results showed that THPH treatment significantly reversed the level of some differential metabolites of serum in the model group, including lysophosphatidylcholine 16:1 and 9-KODE, which were mainly involved in lipid metabolism. It was closely related to the metabolites of probiotics *Lactobacillus reuteri* and *Prevotellaceae* UCG-001, which were involved in the synthesis of fatty acids by activating the free fatty acid receptors (FFAR), thus improving the disorder of lipid metabolism [68,69]. Moreover, THPH markedly upregulated L-glutamic acid, L-histidine, methionine and other metabolites, which were involved in β-alanine metabolism, tyrosine metabolism, arginine and proline metabolism, indicating that THPH improved the disorder of amino acid metabolism of serum in mice with cognitive impairment. Previous studies had shown that the development of amino acid metabolism in host serum largely depend on the changes of gut microbiota, which was consistent with the results of correlation analysis in our study [70]. In addition, we found other important microbial metabolites significantly increased after THPH treatment, including taurine and choline. Choline, the precursor for the synthesis of acetylcholine in cholinergic system, participated in lipid metabolism and amino acid metabolism, which entered into the brain through the blood-brain barrier to regulate the transmission of neural signals and played roles in neuroprotection. The significant increase in serum choline level was deduced from the upregulation of methionine (methyl-donors), which was regulated by gut microbiota and ingested low MW peptides [71,72,73]. Meanwhile, methionine directly generated taurine by trans-sulfurization. Another, methionine further promoted the synthesis of taurine by enhancing the synthesis of cysteine through transmethylation in methionine cycle [74]. As an amino acid neurotransmitter, taurine may prevent mitochondrial dysfunction by regulating osmotic pressure after crossing the blood-brain barrier [75]. It also regulated the generation rate of ROS in brain cells to alleviate the damage caused by oxidative stress and promoted the proliferation of brain cells [76]. These findings indicated that THPH diet restored a variety of serum metabolic disorders caused by scopolamine, especially amino acids metabolism and lipid metabolism, which were attributed to the restructuring of gut microbiota, and finally affected cholinergic system and oxidative stress process in the brain. In short, THPH diet intervention improved brain functions via restructuring gut microbiota and altering microbial metabolites.

Overall, our current research showed that THPH supplementation improved cognitive impairment in mice induced by scopolamine. This was concluded from mice behavior, histopathological changes in the brain and biochemical indicators. Further results suggested that the mechanism mediated by THPH treatment was attributed to the remodeling of the gut microbiota and the changing of serum metabolites. Specifically, THPH increased the abundance of beneficial bacteria and decreased the abundance of harmful bacteria. Moreover, the various disordered metabolic pathways such as amino acid metabolism and lipid metabolism in serum were improved. Meanwhile, THPH also maintained the stability of the cholinergic system in the brain and inhibited the oxidative stress in the nervous system through the microbiota–gut-brain axis. Therefore, supplementation of THPH could be used to prevent cognitive decline as an easy-to-implement nutritional strategy based on maintaining the stability of the gut–microbiota–brain axis. Further studies should evaluate the targeting effect of specific active ingredients of THPH on microbiota-metabolite genes to further alleviate the development of AD.

## Figures and Tables

**Table 1:** Molecular Weight Distribution in THPH.

MW Range	Percentage of Peak Area (%)
>10 kDa	0.02
5–10 kDa	0.27
3–5 kDa	1.19
1–3 kDa	12.59
500–1000 Da	24.12
<500 Da	61.81
(<3 kDa)	(98.52)

**Table 2:** Amino Acid Composition of THPH.

Amino Acid	Contents of Amino Acids (g/100 g)
Aspartate (Asp)	7.02
Glutamate (Glu)	13.24
Serine (Ser)	2.90
Histidine (His)	1.07
Glycine (Gly)	13.00
Threonine (Thr)	2.99
Arginine (Arg)	5.88
Alanine (Ala)	7.05
Tyrosine (Tyr)	1.10
Cystine (Cys-s)	0.08
Valine (Val)	2.40
Methionine (Met)	1.50
Phenylalanine (Phe)	2.38
Isoleucine (Ile)	2.55
Leucine (Leu)	4.46
Lysine (Lys)	4.06
Proline (Pro)	8.14
HAA ^1^	29.58

^1^ Hydrophobic amino acids (HAA): Ala, Val, Met, Cys, Ile, Leu, Tyr, Phe, and Pro.

## Data Availability

All raw data supporting reported results is available from authors upon request.

## Supplementary Materials

The following are available online: https://www.mdpi.com/article/10.3390/foods10123129/s1. Figure S1: Heatmap of functional pathways on gut microbiota in different groups. (A) Heatmap analysis on KEGG functional pathway at level 1 by using Tax4Fun. (B) Heatmap analysis on KEGG functional pathway at level 2 by using Tax4Fun. KEGG, Kyoto Encyclopedia of Genes and Genomes; Figure S2: Heatmap of differential metabolites of mice serum in the model and control group. (A,B) Heatmap analysis on differential metabolites between the control group and the model group in positive and negative ion mode. Figure S3: Heatmap of differential metabolites of mice serum in the THPH and model group. (A,B) Heatmap analysis on differential metabolites between the THPH group and the model group in positive and negative ion mode; Figure S4: The biological metabolic pathways on differential metabolites of mice serum in different groups. (A,B) Bubble diagrams of KEGG pathway enrichment analysis on differential metabolites between the model group and the control group in positive and negative ion mode. (C,D) Bubble diagrams of KEGG pathway enrichment analysis on differential metabolites between the THPH group and the model group in positive and negative ion mode.
