# Supplementary material for: Tilapia Head Protein Hydrolysate Attenuates Scopolamine-Induced Cognitive Impairment through the Gut-Brain Axis in Mice
Source: Foods. 2021 Dec 17;10(12):3129. doi: 10.3390/foods10123129 (PMC8701847; doi:10.3390/foods10123129)
Supplement: Supplementary file 1 [file foods-10-03129-s001.zip › foods-1483062-supplementary.pdf]

**Supplementary Materials**

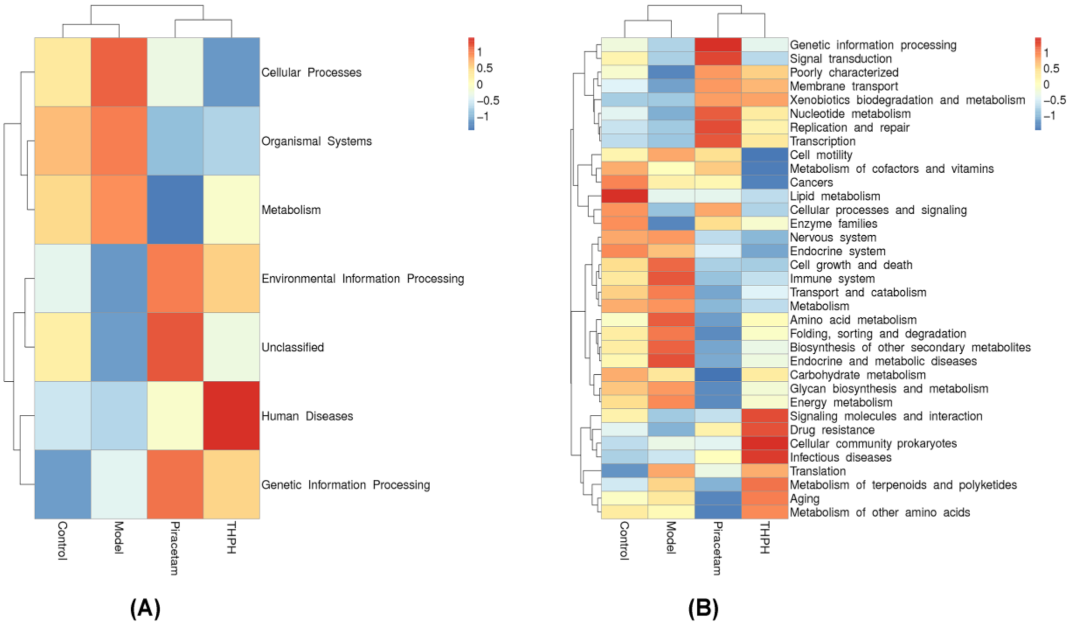


**Figure S1** Heatmap of functional pathways on gut microbiota in different groups. (A) Heatmap analysis on KEGG functional pathway at level 1 by using Tax4Fun. (B) Heatmap analysis on KEGG functional pathway at level 2 by using Tax4Fun. KEGG, Kyoto Encyclopedia of Genes and Genomes.

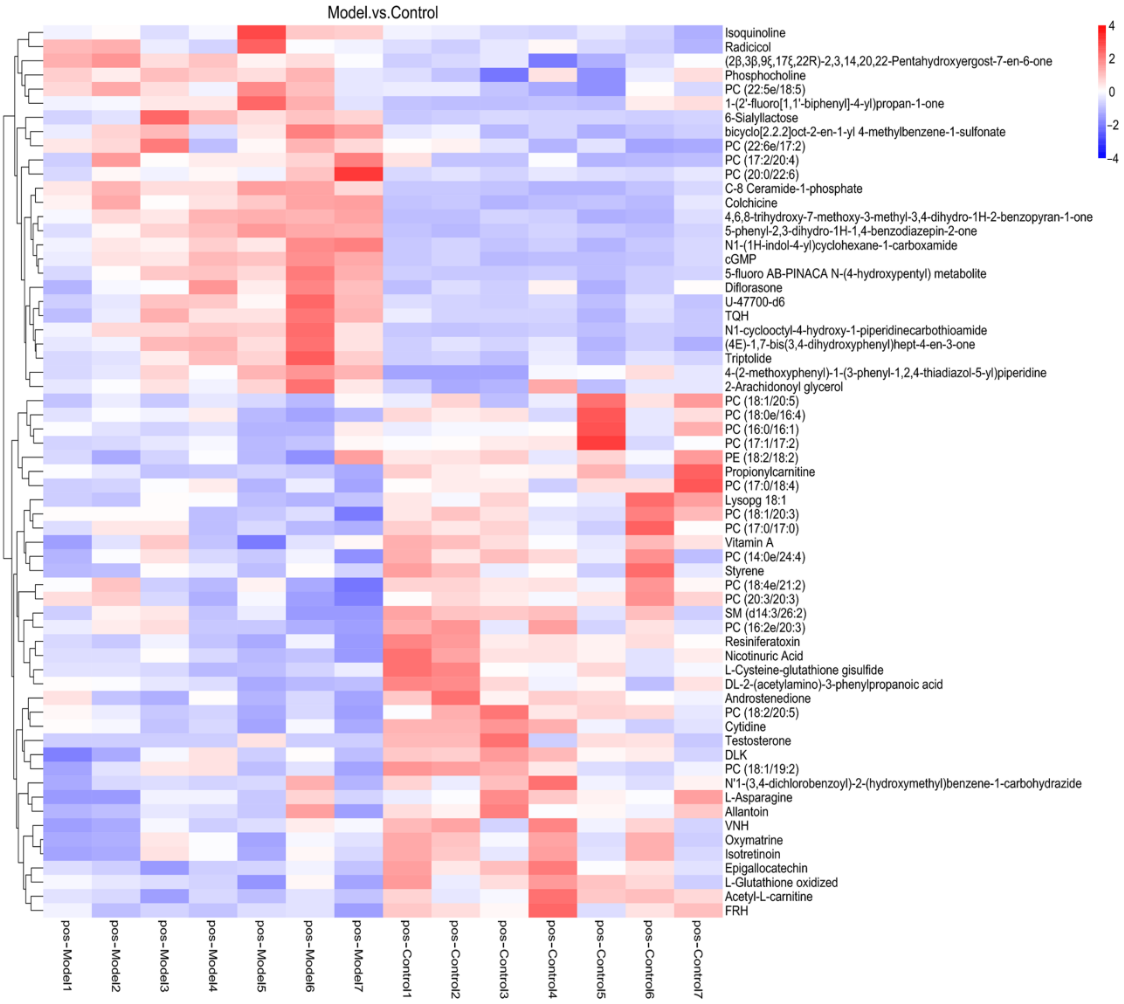


**(A)**

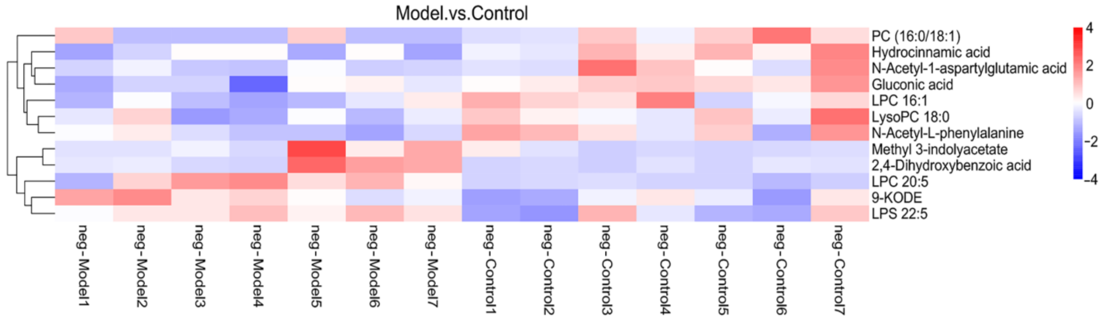


**(B)**

**Figure S2** Heatmap of differential metabolites of mice serum in the model and control group. (A) (B) Heatmap analysis on differential metabolites between the control group and the model group in positive and negative ion mode.

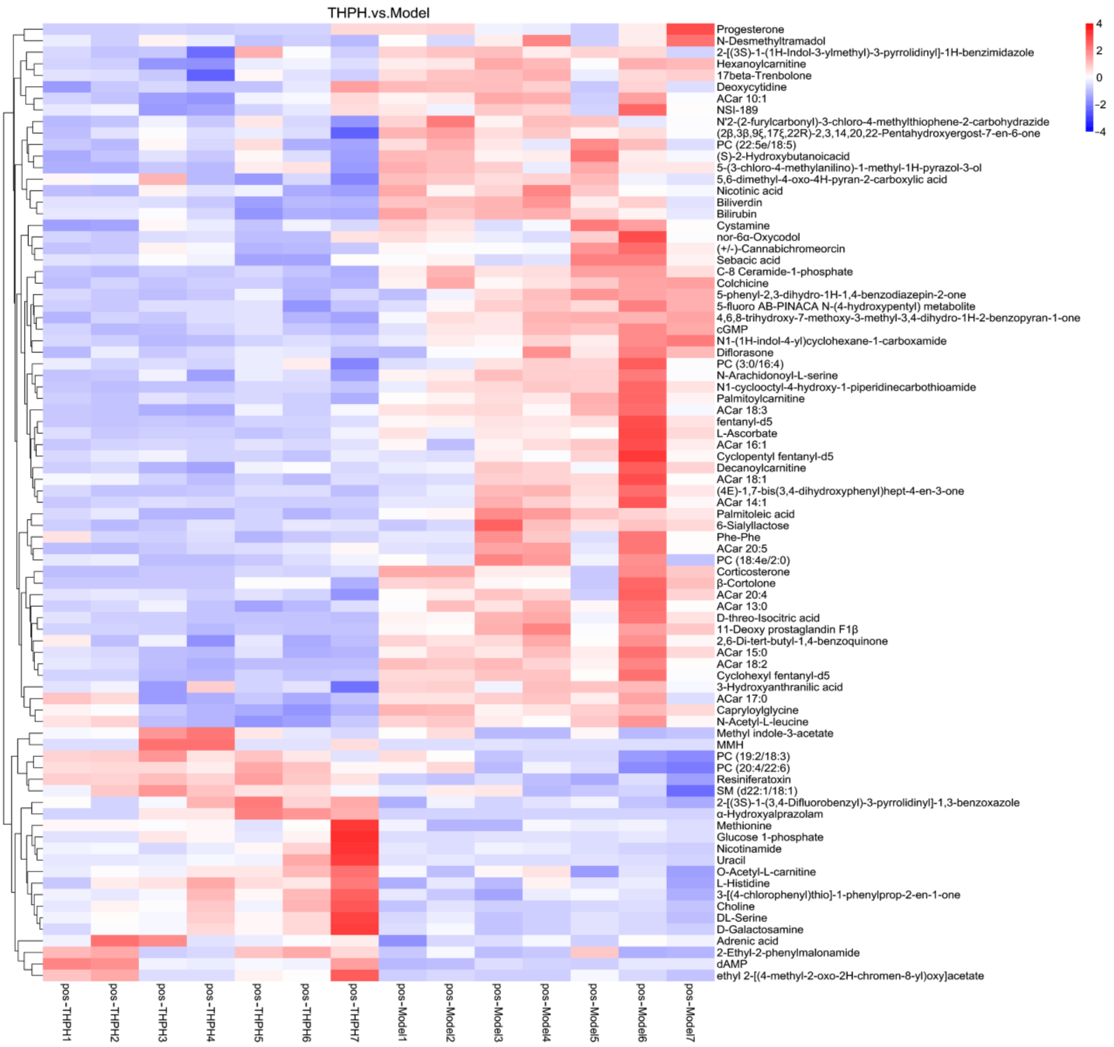
THPH.vs.Model
Progesterone
N-Desmethyltramadol
2-[(3S)-1-(1H-Indol-3-ylmethyl)-3-pyrrolidinyl]-1H-benzimidazole
Hexanoylcarnitine
17beta-Trenbolone
Deoxycytidine
ACar 10:1
NSI-189
N'2-(2-furylcarbonyl)-3-chloro-4-methylthiophene-2-carbohydrazide
(2β,3β,9ξ,17ξ,22R)-2,3,14,20,22-Pentahydroxyergost-7-en-6-one
PC (22:5e/18:5)
(S)-2-Hydroxybutanoicacid
5-(3-chloro-4-methylanilino)-1-methyl-1H-pyrazol-3-ol
5,6-dimethyl-4-oxo-4H-pyran-2-carboxylic acid
Nicotinic acid
Biliverdin
Bilirubin
Cystamine
nor-6α-Oxycodol
(+/-)-Cannabichromeorcin
Sebacic acid
C-8 Ceramide-1-phosphate
Colchicine
5-phenyl-2,3-dihydro-1H-1,4-benzodiazepin-2-one
5-fluoro AB-PINACA N-(4-hydroxypentyl) metabolite
4,6,8-trihydroxy-7-methoxy-3-methyl-3,4-dihydro-1H-2-benzopyran-1-one
cGMP
N1-(1H-indol-4-yl)cyclohexane-1-carboxamide
Diflorasone
PC (3:0/16:4)
N-Arachidonoyl-L-serine
N1-cyclooctyl-4-hydroxy-1-piperidinecarbothioamide
Palmitoylcarnitine
ACar 18:3
fentanyl-d5
L-Ascorbate
ACar 16:1
Cyclopentyl fentanyl-d5
Decanoylcarnitine
ACar 18:1
(4E)-1,7-bis(3,4-dihydroxyphenyl)hept-4-en-3-one
ACar 14:1
Palmitoleic acid
6-Sialyllactose
Phe-Phe
ACar 20:5
PC (18:4e/2:0)
Corticosterone
β-Cortolone
ACar 20:4
ACar 13:0
D-threo-Isocitric acid
11-Deoxy prostaglandin F1β
2,6-Di-tert-butyl-1,4-benzoquinone
ACar 15:0
ACar 18:2
Cyclohexyl fentanyl-d5
3-Hydroxyanthranilic acid
ACar 17:0
Capryloylglycine
N-Acetyl-L-leucine
Methyl indole-3-acetate
MMH
PC (19:2/18:3)
PC (20:4/22:6)
Resiniferatoxin
SM (d22:1/18:1)
2-[(3S)-1-(3,4-Difluorobenzyl)-3-pyrrolidinyl]-1,3-benzoxazole
α-Hydroxyalprazolam
Methionine
Glucose 1-phosphate
Nicotinamide
Uracil
O-Acetyl-L-carnitine
L-Histidine
3-[(4-chlorophenyl)thio]-1-phenylprop-2-en-1-one
Choline
DL-Serine
D-Galactosamine
Adrenic acid
2-Ethyl-2-phenylmalonamide
dAMP
ethyl 2-[(4-methyl-2-oxo-2H-chromen-8-yl)oxy]acetate
pos-THPH1
pos-THPH2
pos-THPH3
pos-THPH4
pos-THPH5
pos-THPH6
pos-THPH7
pos-Model1
pos-Model2
pos-Model3
pos-Model4
pos-Model5
pos-Model6
pos-Model7
4
2
0
−2
−4

**(A)**

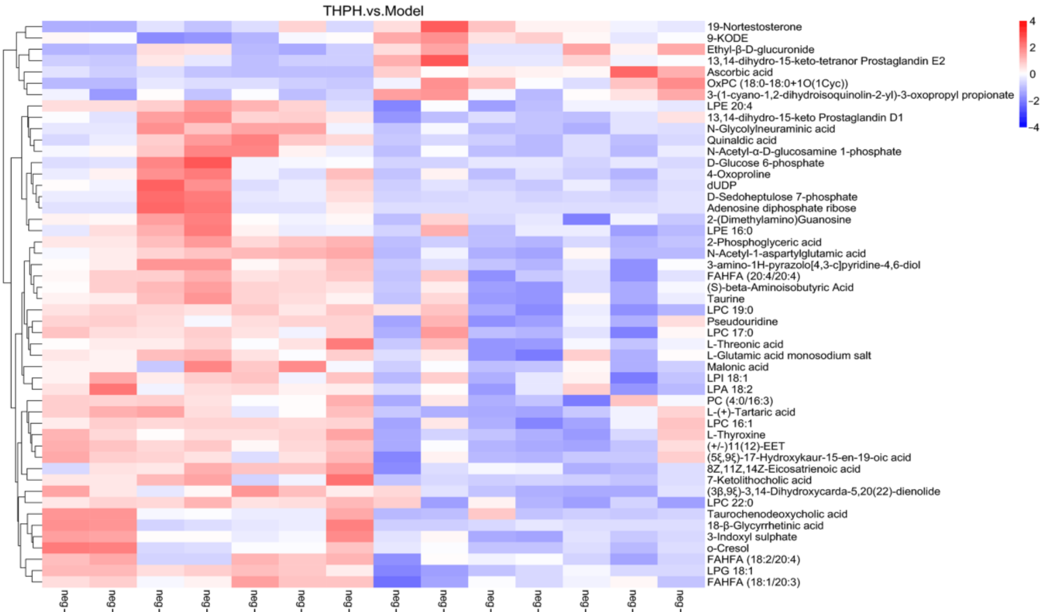
THPH.vs.Model
19-Nortestosterone
9-KODE
Ethyl-β-D-glucuronide
13,14-dihydro-15-keto-tetranor Prostaglandin E2
Ascorbic acid
OxPC (18:0-18:0+1O(1Cyc))
3-(1-cyano-1,2-dihydroisoquinolin-2-yl)-3-oxopropyl propionate
LPE 20:4
13,14-dihydro-15-keto Prostaglandin D1
N-Glycolylneuraminic acid
Quinaldic acid
N-Acetyl-α-D-glucosamine 1-phosphate
D-Glucose 6-phosphate
4-Oxoproline
dUDP
D-Sedoheptulose 7-phosphate
Adenosine diphosphate ribose
2-(Dimethylamino)Guanosine
LPE 16:0
2-Phosphoglyceric acid
N-Acetyl-1-aspartylglutamic acid
3-amino-1H-pyrazolo[4,3-c]pyridine-4,6-diol
FAHFA (20:4/20:4)
(S)-beta-Aminoisobutyric Acid
Taurine
LPC 19:0
Pseudouridine
LPC 17:0
L-Threonic acid
L-Glutamic acid monosodium salt
Malonic acid
LPI 18:1
LPA 18:2
PC (4:0/16:3)
L-(+)-Tartaric acid
LPC 16:1
L-Thyroxine
(+/-)11(12)-EET
(5ξ,9ξ)-17-Hydroxykaur-15-en-19-oic acid
8Z,11Z,14Z-Eicosatrienoic acid
7-Ketolithocholic acid
(3β,9ξ)-3,14-Dihydroxycarda-5,20(22)-dienolide
LPC 22:0
Taurochenodeoxycholic acid
18-β-Glycyrrhetinic acid
3-Indoxyl sulphate
o-Cresol
FAHFA (18:2/20:4)
LPG 18:1
FAHFA (18:1/20:3)
neg-THPH1
neg-THPH2
neg-THPH3
neg-THPH4
neg-THPH5
neg-THPH6
neg-THPH7
neg-Model1
neg-Model2
neg-Model3
neg-Model4
neg-Model5
neg-Model6
neg-Model7
4
2
0
−2
−4

**(B)**

**Figure S3** Heatmap of differential metabolites of mice serum in the THPH and model group. (A) (B) Heatmap analysis on differential metabolites between the THPH group and the model group in positive and negative ion mode.

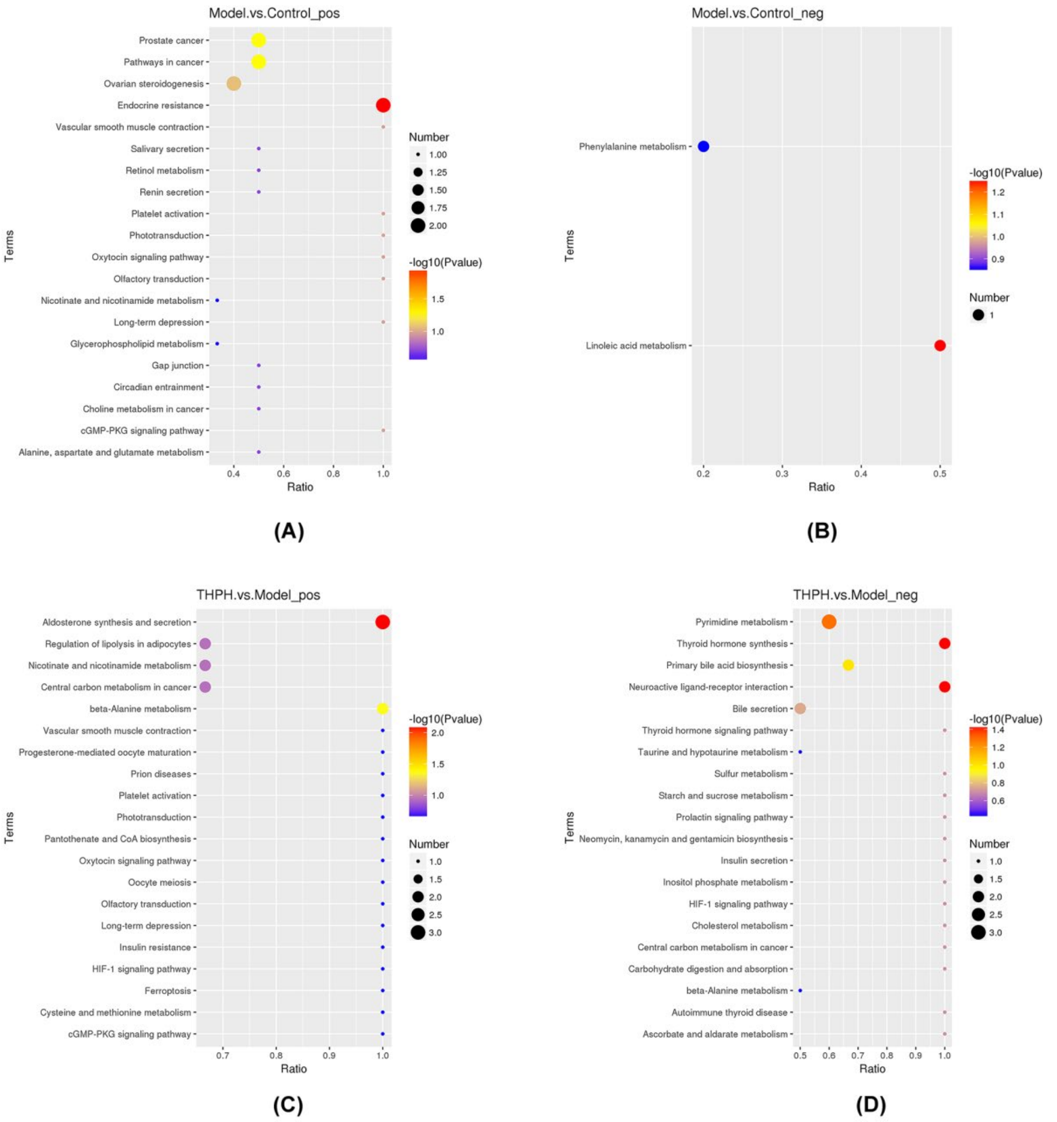


**Figure S4** The biological metabolic pathways on differential metabolites of mice serum in different groups. (A) (B) Bubble diagrams of KEGG pathway enrichment analysis on differential metabolites between the model group and the control group in positive and negative ion mode. (C) (D) Bubble diagrams of KEGG pathway

enrichment analysis on differential metabolites between the THPH group and the model group in positive and negative ion mode.
